# A Dual-Pathway Perspective on Food Choices in Adolescents: The Role of Loss of Control Over Eating

**DOI:** 10.3389/fpsyg.2021.630000

**Published:** 2021-03-31

**Authors:** Eva Van Malderen, Eva Kemps, Laurence Claes, Sandra Verbeken, Lien Goossens

**Affiliations:** ^1^Department of Developmental, Personality and Social Psychology, Ghent University, Ghent, Belgium; ^2^School of Psychology, Flinders University, Adelaide, SA, Australia; ^3^Faculty of Psychology and Educational Sciences, KU Leuven, Leuven, Belgium; ^4^Faculty of Medicine and Health Sciences, University of Antwerp, Antwerp, Belgium

**Keywords:** adolescents, food choices, dual-pathway, inhibitory control, attentional bias, loss of control over eating

## Abstract

**Introduction:**

One in three adolescents frequently consume unhealthy snacks, which is associated with negative developmental outcomes. To date, it remains unclear how intrapersonal factors account for food choices in adolescents. Guided by the dual-pathway model, the current study aimed to: (1) examine the joint contribution of inhibitory control and attentional bias in predicting unhealthy food choices in adolescents, and (2) determine whether this mechanism is more pronounced in adolescents who experience loss of control over eating (LOC).

**Materials and Methods:**

A community sample of 80 adolescents (65% female; 10–17 years old, *M*_age_ = 13.28, *SD* = 1.94) was recruited. Based on a self-report questionnaire, 28.7% of this sample reported at least one episode of LOC over the past month. Food choice was assessed using a computerized food choice task. Both inhibitory control and attentional bias were measured with behavioral tasks (go/no-go and dot probe task, respectively). Binary logistic regressions were conducted to address the research questions.

**Results:**

Inhibitory control and attentional bias did not significantly interact to predict unhealthy food choices. However, there was a significant three-way interaction between inhibitory control, attentional bias and LOC. For adolescents without LOC, the combination of poor inhibitory control and low attentional bias was significantly associated with unhealthy food choice. Surprisingly, for adolescents with LOC, there was no significant association between unhealthy food choice and inhibitory control or attentional bias.

**Discussion:**

Dual-pathway processes do not seem to add to the explanation of food choice behavior for adolescents with LOC. For adolescents who do not experience LOC, those with poor inhibitory control combined with low attentional bias might be at particular risk for making unhealthy food choices.

## Introduction

### Food Choices in Adolescents

The daily consumption of unhealthy snacks is common among adolescents, with prevalence rates up to 23% in Europe (Inchley et al., 2007) and 27% in Flanders (Matthys et al., 2003). Importantly, snacking is responsible for 20–24% of the total energy intake in this age group (De Cock et al., 2016). Unhealthy snacking has been found to be associated with negative physical (e.g., increased risk of overweight and obesity and related medical morbidities such as cardiovascular diseases) and psychosocial outcomes (e.g., depression, poor academic performance) (WHO, 2003; Gopinath et al., 2014; Khalid et al., 2016; Chikwere, 2019). On the contrary, healthy eating can be considered a protective factor due to its associations with a wide range of positive health outcomes (Haines et al., 2019). Consequently, tackling unhealthy eating and improving healthy eating are key public health priorities. Because adolescence is a period when individuals gain more autonomy from parents, and thus assume greater responsibility for their own food choices and behavior, this developmental period is of particular importance to study eating behavior in general, and food choices in particular (Steinberg, 2005). Moreover, adolescence is a period of increasing cognitive maturation, characterized by high reactivity to the environment (e.g., attention for rewarding stimuli) and further development of behavioral regulatory skills (e.g., inhibiting responses) (Crone et al., 2016). Therefore, a more thorough understanding of the underlying mechanisms that drive food choices in adolescents is warranted.

### Dual-Pathway Perspective on Food Choices

Transactional models assume that our everyday behavior, such as our eating behavior, is driven by a complex interplay of intrapersonal (e.g., self-regulation) and interpersonal (e.g., food environment) factors (Braet et al., 2014; Lewis, 2014). Notwithstanding the impact of interpersonal factors on eating behavior (for example see Downs and Demmler, 2020), intrapersonal factors are an important target of eating behavior interventions (for example see Köster, 2009; Luis-Ruiz et al., 2020).

A comprehensive theoretical account of the intrapersonal determinants of eating behavior is the dual-pathway perspective (Strack and Deutsch, 2004). This perspective proposes that eating behavior is governed by two interacting systems: *regulatory processes* which are slow and deliberate (e.g., inhibitory control) and *reactive processes* which are fast and effortless (e.g., attentional bias). According to this perspective, unhealthy food choices may be the result of an imbalance between immature regulatory processes (e.g., poor inhibitory control when confronted with palatable food) coupled with strong reactive processes (e.g., automatic attention toward palatable food in the environment) (Steinberg et al., 2018).

Guided by the dual-pathway perspective, researchers have recently found evidence for the *joint contribution* of regulatory and reactive processes to eating behavior. For example, Kakoschke et al. (2015) reported that the combination of poor regulatory processes and strong reactive processes predicted unhealthy food intake from a taste test in adults. Similarly in adolescents, poor regulatory processes coupled with strong reactive processes have been associated with self-reported unhealthy food intake (Stok et al., 2015). In the same vein, both Van Malderen et al. (2020) and Booth et al. (2018) provided evidence in support of a dual-pathway account of self-reported uncontrolled eating among adolescents.

Although the dual-pathway perspective states that both types of processes interact to predict eating behavior, there is already plenty of evidence for the *independent role* of either *poor inhibitory control* (e.g., Nederkoorn et al., 2012; Byrne et al., 2020a) or *high attentional bias* toward food (e.g., Werthmann et al., 2011; Yokum et al., 2011; Folkvord et al., 2015) in predicting eating behavior among adolescents (e.g., uncontrolled eating, unhealthy snacking), whereas studies that investigate the combination of these processes are scarce.

Furthermore, studies have generally not specifically focused on *food choices* as an outcome variable, but rather on broader outcome variables such as food consumption or uncontrolled eating. However, to gain a more comprehensive understanding of the determinants of unhealthy eating behavior, a crucial first step is to identify the factors that contribute to unhealthy food choices in adolescents.

### Role of Loss of Control Over Eating in Food Choice

It is unclear whether the central assumptions of the dual-pathway perspective apply to food choices in all adolescents or whether these are particularly pronounced in those who experience early signs of eating-disordered behavior. Specifically, it has been shown that one in three adolescents report *loss of control over eating (LOC)*, which can be defined as the experience of lack of control while eating (He et al., 2016; Van Malderen et al., 2020). LOC is a central feature of binge eating and research has demonstrated that adolescents who experience LOC are at an increased risk for developing negative health outcomes such as overweight and obesity (Goossens et al., 2009a; Shomaker et al., 2010; Tanofsky-Kraff et al., 2011). Moreover, longitudinal research has shown that one episode of LOC in adolescents may be considered an early sign of eating disordered behavior given its prospective value for the development of clinical eating disorders (e.g., Bulimia Nervosa) and other types of psychopathology (e.g., depression, addiction) (Tanofsky-Kraff et al., 2011; Herpertz-Dahlmann et al., 2015), thereby emphasizing its clinical significance. Importantly, previous research has provided evidence for the dual-pathway perspective in predicting LOC among adolescents (Booth et al., 2018; Van Malderen et al., 2020). In addition, it has been shown that adolescents who experience LOC eat more palatable food and make more unhealthy food choices (Dalton et al., 2013; Ng and Davis, 2013; Byrne et al., 2020b). Both findings highlight the importance of taking into account *how* one feels while eating (i.e., food experience) alongside *what* one chooses to eat (i.e., food choices). Thus, in investigating food choice behavior in adolescents from a dual-pathway perspective, it may be important to include LOC as a moderator to distinguish adolescents with LOC from those without LOC.

### Current Study

The current study aimed to investigate the underlying mechanisms of food choice behavior in adolescents. To this end, the study addressed two main research questions. First, based on the dual-pathway perspective, we examined the interaction between regulatory (i.e., inhibitory control) and reactive (i.e., attentional bias) processes in predicting food choice in adolescents. Guided by previous empirical evidence (e.g., Kakoschke et al., 2015; Stok et al., 2015; Booth et al., 2018; Van Malderen et al., 2020), we expected that the combination of poor inhibitory control and high attentional bias would be associated with the greatest risk of unhealthy food choices among adolescents. Second, to determine whether dual-pathway assumptions apply to food choice behavior of all adolescents or may be more pronounced in those with early signs of eating-disordered behavior (for example see Herpertz-Dahlmann et al., 2015), LOC was included as an additional moderator. As LOC has previously been associated with the dual-pathway processes (Booth et al., 2018; Van Malderen et al., 2020), as well as with unhealthy food choices (Dalton et al., 2013; Ng and Davis, 2013; Byrne et al., 2020b), it was hypothesized that the interaction between regulatory and reactive processes would be more pronounced in adolescents with LOC compared to those without LOC.

## Materials and Methods

### Participants and Procedure

The sample consisted of 80 participants, recruited from the general population. Participants were contacted by 3rd year psychology students (in the context of a practical course). Each student was instructed to recruit two participants between 10 and 18 years old (there were no other in- or exclusion criteria). We based this age range on the commonly used definition of adolescence in the literature (i.e., the transitional period between childhood and adulthood) (Sawyer et al., 2018). In the final sample, participants were between 10 and 17 years old (*M*_age_ = 13.28, *SD* = 1.94) and 65% (*N* = 52) of the sample was female. Data collection occurred during a home visit, and consisted of two parts. First, participants were presented with several online questionnaires. Second, participants completed two computer tasks the order of which was counterbalanced (i.e., go/no-go task and dot probe task), followed by a computerized food choice task (see section “Materials”). The total duration of each home visit was approximately 2 h. All adolescents and their parents signed an active informed consent and the entire study protocol was approved by the Faculty Ethics Committee. In the informed consent, the study was described as investigating risk and protective factors for the development of psychological problems during adolescence. No incentives were provided for participation. The study was part of a broader project on eating behavior among adolescents and some of the data have been reported previously (Van Malderen et al., 2019, 2020). The focus of the current study was on the dual-pathway predictors of food choice among adolescents, whereas the other studies focused on the role of affectivity (Van Malderen et al., 2019) and self-regulation (Van Malderen et al., 2020) in loss of control over eating in adolescents. Consequently, only participants who had completed the food choice task (*N* = 80) were included in the current sample.

### Materials

#### Control Variables

Participants self-reported their age and gender. During the home visit, height and weight were objectively measured (using a tape measure and scales). An adjusted body mass index was calculated {[actual body mass index (kg/m^2^)/percentile 50 of body mass index for age and gender] × 100} (Roelants and Hauspie, 2004; Rolland-Cachera et al., 2015). Because food choice may be influenced by age, gender, and adjusted body mass index, these were included as control variables in all analyses (Manippa et al., 2017; Andrade et al., 2019; Perrar et al., 2020).

#### Food Choice

Food choice was assessed with a computerized *food choice task* (see Veling et al., 2013). In this task, participants were presented with a 4 × 4 square grid with 16 pictures of snacks on a computer screen and asked to select eight items that they would like to take home. There were eight healthy snacks (i.e., carrots, gingerbread, health bars, fruit salad, apple, muesli bars, crackers, rice cake) and eight unhealthy snacks (i.e., potato chips, chocolate, muffin, salted nuts, cheese balls, M&M’s, chocolate chip cookies, cookies). The pictures for this task were derived from Veling et al. (2013) who validated these in terms of palatability and healthiness in an independent sample of participants. Importantly, the food pictures in this task represented the same broad categories as the food pictures of the tasks that capture dual-pathway processes (see below). The time limit for making food selections was 15 s. Following previous research (e.g., Furst et al., 1996; Kakoschke et al., 2017), the outcome measure of interest was the first snack item chosen (0 = healthy snack, 1 = unhealthy snack). This ensured that an “automatic” decision was captured.

#### Dual-Pathway Processes

The *“go/no-go task” (GNG)* was used as a measure of *regulatory processing*, and more specifically *inhibitory control* (see Kakoschke et al., 2015). In this task, participants were presented with two blocks of 160 trials. Each trial had a duration of 1500 ms in which a picture was shown coupled with either a “go” cue (e.g., the letter “p”) to which participants responded by pressing the space bar, or a “no-go” cue (e.g., the letter “f”) which signaled that participants should withhold their response. The “go” and “no-go” cues appeared randomly at one of the four corners of the picture. Both types of cues appeared equally often during the task and were counterbalanced (i.e., for some participants the “go” cue was the letter “f” and for others it was the letter “p”). The pictures were taken from the food-pics database of Blechert et al. (2014). Specifically, the pictures consisted of images of 20 palatable foods (e.g., chips, chocolate) and 20 non-foods (i.e., animals rated to be of similar appeal) (e.g., giraffe, butterfly). The outcome measure was the number of commission errors (i.e., CE; space bar pressed in response to a “no-go” cue) (e.g., see Meule and Kübler, 2014). A higher number of commission errors on food pictures (CE_food_) reflects poorer inhibitory control toward food.

To measure *reactive processing*, and specifically *attentional bias*, the *“dot probe task” (DP)* was used (see Kakoschke et al., 2014). The task consisted of 258 trials. Each trial commenced with a fixation cross presented in the middle of the screen (for 500 ms), followed by two pictures presented simultaneously on the left and right hand sides of the screen (also for 500 ms). Next, a dot (probe) was presented in the location of one of the two pictures. The participant’s task was to indicate as quickly as possible whether the dot (probe) appeared on the left or right hand side of the screen by pressing a key on an AZERTY-keyboard (“W” and “N,” respectively). Again, the pictures were sourced from the food-pics database of Blechert et al. (2014). Two pairs of stimuli were used: food versus neutral non-food (32 experimental pairs) and neutral non-food versus neutral non-food (16 control pairs). For the experimental pairs, household objects were chosen as the neutral non-food category. Only these pairs were used to calculate an attentional bias score for inclusion in the analyses. The stimuli for the control pairs consisted of animals, because like food, animals are overall appealing. Household objects and animals are commonly used neutral non-food categories in attentional bias research (e.g., Kemps et al., 2014; Liu et al., 2019). The pictures in each pair were matched on color and shape. Picture pairs were presented in a new random order for each participant. The dot (probe) appeared equally often on both sides of the screen. The outcome measure was reaction time (RT; in milliseconds). Attentional bias scores (AB) were computed from the experimental trials by subtracting the RT on trials where the probes replaced the food pictures from the RT on trials where the probes replaced the neutral non-food pictures. A positive score reflects an attentional bias toward food pictures and a negative score an attentional bias away from food pictures.

#### Loss of Control Over Eating (LOC)

The experience of loss of control over eating (LOC) was assessed with the Dutch translation and adaptation of the “Children’s Eating Disorder Examination Questionnaire” (ChEDE-Q; Fairburn and Beglin, 1994; Decaluwé and Braet, 1999). This self-report questionnaire consists of four underlying subscales (i.e., restrictive eating, concerns about eating, weight, and shape), and in addition assesses different types of uncontrolled eating episodes (i.e., objective binge eating, subjective binge eating). For the current study, only the questions assessing uncontrolled eating episodes were used. Participants were first asked if they had experienced that type of eating episode over the past month (yes/no). If yes, the total number of such episodes over the past month was determined. The variable of interest was whether or not participants had experienced at least one episode of uncontrolled eating over the past month (0 = no LOC episode over the past month, 1 = at least one LOC episode over the past month). This operationalization (i.e., one episode over a 1-month time frame) is in line with other such studies among adolescents in the general population (e.g., Tanofsky-Kraff et al., 2011, 2020; Kelly et al., 2016). Previous research has shown that the ChEDE-Q is a valid and reliable measure of LOC in adolescents (Decaluwé et al., 2003; Van Durme et al., 2015).

### Statistical Analysis

To test the interaction between regulatory (i.e., inhibitory control) and reactive (i.e., attentional bias) processing in predicting food choice, a binary logistic regression was conducted. First, food choice was entered as a categorical dependent variable (0 = healthy snack, 1 = unhealthy snack). Second, age, gender and adjusted body mass index were entered as control variables. Third, the main effects of inhibitory control (i.e., CE_food_), attentional bias (i.e., AB), and their interaction (i.e., CE_food_ × AB) were included as independent variables.

To investigate whether the dual-pathway perspective applies to food choice in adolescents in general or is more pronounced in those with existing disturbed eating behavior, an additional binary logistic regression analysis was performed. This analysis was identical to the first regression, but with LOC included as an additional categorical moderator (0 = no LOC episode over the past month, 1 = at least one LOC episode over the past month) in the interaction term that was added in the last step (i.e., CE_food_ × AB × LOC). To ascertain the robustness of any interaction effect, the analyses were also performed without covariates (i.e., age, gender, and adjusted body mass index).

Only the full logistic regression models (including the control variables and all independent variables) are displayed (see Tables 2, 3) in the Results. Significant interactions were interpreted by comparing the means between the different groups using independent sample *t*-tests. An alpha value of *p* ≤ 0.05 was used to determine statistically significant effects and odds ratios (OR) were reported as effect sizes for all analyses. The analyses were conducted with SPSS version 24.0.

## Results

### Descriptive Statistics

The mean adjusted body mass index of the sample was 100.32 (*SD* = 17.41), and ranged from adolescents having underweight (minimum = 58.62) to adolescents having obesity (maximum = 163.47). Specifically, 11% of the sample was classified as having underweight (adjusted body mass index ≤85), 79% as having a normal weight (85 <adjusted body mass index <120), 5% as having overweight (120 <adjusted body mass index <140), and 5% as having obesity (adjusted body mass index ≥140).

Mean number of commission errors on the food pictures of the go/no-go task was low (*M* = 3.10, *SD* = 4.67), reflecting good overall inhibitory control capacities toward food pictures. However, there was a large degree of variability in the number of commission errors, ranging from 0 to 37 (also see Kakoschke et al., 2015; Van Malderen et al., 2020). The mean attentional bias score from the dot probe task was positive (*M* = 1.07, *SD* = 21.51). However, a one sample *t*-test showed that this was not significantly different from zero, *t*(79) = 0.44, *p* = 0.658. The standard deviation was again large, indicating substantial variability across participants (ranging from −50.75 to 61.96). In total, 28.7% (*N* = 23) of participants reported at least one episode of LOC over the past month (according the ChEDE-Q; Fairburn and Beglin, 1994; Decaluwé and Braet, 1999). Among those, the number of episodes ranged from 1 to 20 (*Median* = 2.00, *Mean* = 4.04, *SD* = 4.80). Most adolescents in that group reported 1 (21.7% or *N* = 5) or 2 episodes of LOC (34.9% or *N* = 8). Furthermore, 13.1% adolescents (*N* = 3) reported 4 episodes of LOC, 13.1% (*N* = 3) 3 episodes, and 4.3% (*N* = 1) 5, 11, 15, and 20 episodes of LOC over the past month. In the food choice task, 48.8% (*N* = 39) of participants chose an unhealthy food first. Table 1 gives an overview of all sample characteristics and correlations between the variables of interest.

**TABLE 1:** Descriptive statistics and correlations.

Total sample	*N*	*M* (*SD*) or %	Min – Max	LOC^a^	Age	AdjBMI	CE_food_	AB
Gender	80	65% female						
Food choice	80	48.8% unhealthy						
LOC	80	28.7% LOC	1 – 20	1				
Age	80	13.28 (1.94)	10 – 17	0.15	1			
AdjBMI	80	100.32 (17.41)	58.62 – 163.47	0.18	−0.07	1		
CE_food_	80	3.10 (4.67)	0 – 37	−0.02	−0.21	0.04	1	
AB	80	1.07 (21.51)	−50.75 – 61.96	−0.07	0.01	−0.14	−0.08	1

**LOC-Group^b^**	*N*	*M* (*SD*) or %	Min – Max		Age	AdjBMI	CE_food_	AB

Gender	23	69.6% female						
Food choice	23	47.8% unhealthy						
Age	23	13.74 (1.96)	10 – 17		1			
AdjBMI	23	105.16 (18.44)	86.05 – 163.47		−0.09	1		
CE_food_	23	3.78 (7.54)	0 – 37		−0.22	0.10	1	
AB	23	−0.05 (25.39)	−45.06 – 61.96		−0.13	−0.10	0.05	1

**NoLOC-Group^b^**	*N*	*M* (*SD*) or %	Min – Max		Age	AdjBMI	CE_food_	AB

Gender	57	63.2%						
Food choice	57	49.1% unhealthy						
Age	57	13.09 (1.91)	10 – 17		1			
AdjBMI	57	98.37 (16.75)	58.62 – 152.24		−0.10	1		
CE_food_	57	2.82 (2.85)	0 – 12		−0.28*	−0.06	1	
AB	57	1.52 (19.97)	−50.75 – 51.24		−0.09	−0.16	−0.26	1

LOC, Loss of Control over Eating; AdjBMI, Adjusted Body Mass Index; CE_food_, Commission Errors on Food Pictures; AB, Attentional Bias Score. ^a^ These correlations are Spearman’s correlations; all other correlations are Pearson’s correlations. ^b^ There were no significant group differences between the LOC-Group and the NoLOC-Group on any of these variables. *p ≤ 0.050.

### Main Analyses

The first binary logistic regression analysis which tested the interaction between regulatory (i.e., inhibitory control) and reactive (i.e., attentional bias) processing in predicting food choice was not significant [χ*^2^*(6) = 7.16, *p* = 0.307], and revealed no significant main or interaction effects (see Table 2). Without the covariates, the analysis was also not significant [χ*^2^*(3) = 1.15, *p* = 0.766], and revealed no significant main or interaction effects.

**TABLE 2:** Logistic regression analysis: Inhibitory control × attentional bias in predicting unhealthy food choice.

	Wald χ *^2^*	*B (SE)*	*p*	*OR*
Covariates:				
Gender	2.09	0.75 (0.52)	0.149	2.11
Age	2.93	0.22 (0.13)	0.087	1.24
AdjBMI	0.45	−0.01 (0.01)	0.504	0.99
CE_food_	0.09	−0.02 (0.06)	0.759	0.98
AB	0.02	−0.00 (0.02)	0.889	1.00
CE_food_ × AB	0.02	0.00 (0.00)	0.892	1.00
Model test	χ^2^ (6) = 7.16, *p* = 0.307			
−2LL (Nagelkerke R^2^)	103.70 (0.11)			

*OR, Odds Ratio; AdjBMI, Adjusted Body Mass Index; CE_food_, Commission Errors on Food Pictures; AB, Attentional Bias Score.*

**FIGURE 1:**
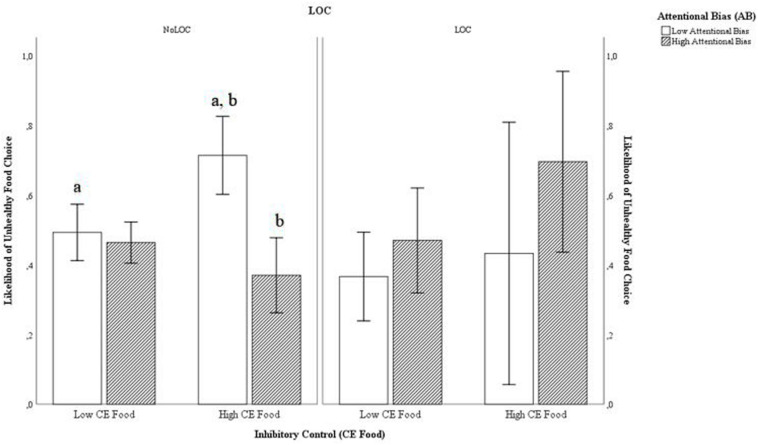
Three-way interaction between inhibitory control (CE_food_), attentional bias (AB), and LOC in predicting unhealthy food choice. CE_food_, Commission Errors on Food Pictures; AB, Attentional Bias Score; LOC, Loss of Control over Eating. a: *t*(29) = 3.46, *p* = 0.002**; b: *t*(22) = –4.49, *p* ≤ 0.001***. Error Bars: 95% confidence interval.

The second binary logistic regression analysis which investigated whether the dual-pathway perspective may be more pronounced in adolescents with LOC was significant [χ*^2^*(7) = 14.27, *p* = 0.047], and revealed a significant three-way interaction (*p* = 0.043) (see Table 3). Without the covariates, the analysis was trend significant [χ*^2^*(4) = 8.85, *p* = 0.065], and again revealed a significant three-way interaction (*p* = 0.035). Specifically, inhibitory control (CE_food_), attentional bias (AB) and LOC significantly interacted to predict unhealthy food choice. Figure 1 shows this three-way interaction. As can be seen, two different patterns emerged for the LOC-group and the NoLOC-group.

**TABLE 3:** Logistic regression analysis: Inhibitory control × attentional bias × LOC in predicting unhealthy food choice.

	Wald χ *^2^*	*B (SE)*	*p*	*OR*
Covariates:				
Gender	1.74	0.70 (0.53)	0.187	2.02
Age	2.38	0.20 (0.13)	0.123	1.22
AdjBMI	0.66	−0.01 (0.02)	0.418	1.01
CE_food_	0.29	−0.04 (0.07)	0.592	1.01
AB	0.15	−0.01 (0.02)	0.703	1.00
CE_food_ × AB	2.00	0.01 (0.01)	0.158	1.01
CE_food_ × AB × LOC	4.09	−0.02 (0.01)	0.043*	1.00
Model test	χ^2^ (7) = 14.27, *p* = 0.047*			
−2LL (Nagelkerke R^2^)	96.59 (0.22)			

OR, Odds Ratio; AdjBMI, Adjusted Body Mass Index; CE_food_, Commission Errors on Food Pictures; AB, Attentional Bias Score; LOC, Loss of Control over Eating. *p ≤ 0.050.

In the *NoLOC-group* (left panel), participants with low AB scores (white bars) were significantly more likely to choose an unhealthy snack first when they also had high levels of CE_food_ (i.e., weaker inhibitory control toward food) (*M* = 0.714, *SD* = 0.202) compared to when they had low levels of CE_food_ (i.e., stronger inhibitory control toward food) (*M* = 0.493, *SD* = 0.152), [*t*(29) = 3.46, *p* = 0.002]. For participants with high AB scores (gray bars), there was no significant association between level of CE_food_ and the likelihood of choosing an unhealthy snack first [*t*(24) = −1.84, *p* = 0.078]. Thus, for participants with high levels of CE_food_ (i.e., weaker inhibitory control toward food), the likelihood of choosing an unhealthy food first was significantly greater for those with low (*M* = 0.714, *SD* = 0.202) compared to high (*M* = 0.370, *SD* = 0.140) AB scores [*t*(22) = −4.49, *p* ≤ 0.001].

In the *LOC-group* (right panel), there was no significant association between level of CE_food_ and the likelihood of choosing an unhealthy snack first, neither for participants with low AB scores (white bars) [*t*(10) = 0.60, *p* = 0.561], nor for those with high AB scores (gray bars) [*t*(9) = 1.61, *p* = 0.142].

## Discussion

Guided by the dual-pathway model, the current study aimed to investigate the interaction between inhibitory control and attentional bias in predicting unhealthy food choices in adolescents. An additional goal was to determine whether this dual-pathway perspective was more pronounced in adolescents with early signs of eating-disordered behavior and specifically those who experience LOC (for example see Herpertz-Dahlmann et al., 2015). By addressing these two research questions, this study sought to contribute to the underlying mechanisms that drive food choice behavior in adolescents.

Based on previous studies and the theoretical dual-pathway perspective (e.g., Kakoschke et al., 2015; Stok et al., 2015; Booth et al., 2018; Van Malderen et al., 2020), we expected a significant interaction between poor inhibitory control and strong attentional bias in predicting unhealthy food choice in adolescents. Contrary to expectation, there was no significant interaction between inhibitory control and attentional bias in predicting food choice (research question 1). However, the inclusion of LOC as an additional moderator revealed a significant three-way interaction between inhibitory control, attentional bias and LOC (research question 2). This shows that the relationship between inhibitory control and attentional bias in predicting unhealthy food choice depends on whether adolescents experienced LOC over the past month. Surprisingly, the direction of this three-way interaction was not in line with dual-pathway predictions and – contrary to our expectations – it was not more pronounced in adolescents with LOC compared to those without LOC (for example see Booth et al., 2018; Byrne et al., 2020b).

In particular, adolescents *without LOC* were more likely to choose an unhealthy food first when they exhibited a combination of *poor inhibitory* control and *low attentional bias*. This result is at odds with dual-pathway predictions that the combination of *poor inhibitory control* and *high attentional bias* would be associated with unhealthy food choices. It also contradicts previous empirical evidence for this dual-pathway perspective in the context of overweight or unhealthy eating in children (e.g., Kemps et al., 2020), adolescents (e.g., Stok et al., 2015), and adults (e.g., Kakoschke et al., 2015). Nevertheless, the current result is in line with other previous observations in adults (Manasse et al., 2015) as well as in adolescents (Van Malderen et al., 2018) which have also found an interaction between poor inhibitory control and low attentional bias. However, it should be noted that all these previous studies focused on overweight or unhealthy eating as the outcome variable and not food choice specifically. In particular, these results seem to indicate that, in adolescents without LOC, the level of attentional bias determines the extent to which inhibitory control capacities contribute to unhealthy food choices. One possible explanation might be that adolescents with *low attentional bias* are less preoccupied with food in general (for example see Brignell et al., 2009; Hardman et al., 2020). When those adolescents also have good inhibitory control capacities, they are then able to go for a healthy food option. However, when this low attentional bias is coupled with poor inhibitory control, their poor regulatory abilities increase their risk of choosing unhealthy food. On the other hand, adolescents with *high attentional bias* are generally more preoccupied with food in the environment, regardless of their inhibitory control capacities (Hendrikse et al., 2015; Kemps et al., 2020). As a result, they are not more nor less likely to make unhealthy food choices depending on their level of inhibitory control. An alternative explanation might be that adolescents with *high attentional bias* deliberately avoid palatable food in the environment and intentionally prefer a healthy snack over an unhealthy one. This adaptive avoidant strategy may explain the finding that, in the NoLOC-group, adolescents with poor inhibitory control are significantly less likely to choose an unhealthy food when they have a strong attentional bias for food. Yet another explanation for the unexpected direction of the interaction in the NoLOC-group, might be that this group is not homogeneous and consists of several important subtypes (e.g., depending on adjusted BMI, temperament, environmental factors) which may have contributed the results (for example see Kubik et al., 2003; Davis and Fox, 2008; De Cock et al., 2016). Future research may distinguish between these possible subtypes by including them as additional moderating variables.

In addition, we hypothesized that poor inhibitory control combined with a strong attentional bias (i.e., dual-pathway perspective) would be particularly associated with unhealthy food choices in adolescents who experience LOC. However, adolescents *with LOC* were not significantly more nor less likely to choose an unhealthy food regardless of their levels of inhibitory control or attentional bias. As an extension of previous evidence for a dual-pathway account of LOC among adolescents (e.g., Booth et al., 2018; Van Malderen et al., 2020), the current findings seem to indicate that this vulnerability does not have additional explanatory value in the context of food choices. A possible explanation might be that the LOC-group is characterized by considerable variation regarding the types of uncontrolled eating episodes. For example, at one particular time one can experience LOC while eating objectively large amounts of food (i.e., objective binge eating episodes) during which it would be expected that unhealthy food would be preferred (for example see Ng and Davis, 2013; Byrne et al., 2020b). In contrast, at other times one can experience LOC while eating an amount of food that is considered to be large only according to the individual but not to others (i.e., subjective binge eating episodes). This subjective type of uncontrolled eating behavior is often accompanied by eating subjectively large amounts of (healthy or unhealthy) foods (Fitzsimmons-Craft et al., 2014). This variation in types of uncontrolled eating behavior might have obscured any effect of inhibitory control and attentional bias on LOC in general.

### Strengths, Limitations and Directions for Future Research

The current study has several important strengths. First, the sample consisted of adolescents. This is a crucial population for studying eating behavior in general and food choices in particular because of the well-known risk of developing eating problems during that age period (Steinberg, 2005). Second, although the dual-pathway perspective emphasizes the importance of investigating the interaction between inhibitory control and attentional bias, most research has focused on these processes individually. The current study adds to the literature specifically by examining their combined contribution. Third, this study focused specifically on food choice instead of broader eating-related outcome variables (e.g., food consumption). This approach enables the further disentanglement of the underlying mechanisms that drive unhealthy food choices, which in turn, contribute to our understanding of unhealthy eating behavior. Finally, the assumptions of the dual-pathway perspective were tested in adolescents with versus those without early signs of eating-disordered behavior, namely LOC (for example see Tanofsky-Kraff et al., 2007; Goossens et al., 2009b; Van Malderen et al., 2020). In making this distinction, the present results may help shed light on whether the theoretical dual-pathway perspective applies to adolescents in general or to particular subgroups.

There are also some noteworthy limitations that should be acknowledged. First, the sample size of the LOC-group was quite small. In addition, the current study did not distinguish between different types of uncontrolled eating episodes (e.g., objective versus subjective binge eating episodes). A larger sample may afford the ability to distinguish between several types of uncontrolled eating episodes and ascertain any clinically relevant effects that were precluded in the current study due to a lack of power. In addition, a larger sample size would allow for the inclusion of other important control variables in the context of food choice behavior, such as educational level or household income (Baumann et al., 2019). Furthermore, the percentage of adolescents with overweight (5%) and obesity (5%) in the present study was lower compared to prevalence rates in the general population (World Health Organization, 2016), limiting the generalizability of the findings.

Second, the study design was cross-sectional, precluding any causal inferences. Moreover, the current design carries the risk of limited ecological validity, making replication in a real-life setting an important goal for future research (e.g., assessing food choices from a real-life food buffet, conducting ecological momentary assessment).

Third, the food choice task was limited in the number and types of food presented. Because food preferences are known to be personal (Birch, 1999; Giese et al., 2015; Robino et al., 2019), a next step would be to personalize the food choice task or use a real-life food buffet.

Fourth, the dual-pathway distinction between regulatory and reactive processes is not entirely clear-cut because these two types of processing cannot be strictly separated (i.e., slow and deliberate regulatory processes versus fast and effortless reactive processes). In particular, it could be argued that the behavioral task that was used to measure regulatory processing (i.e., go/no-go task) not only assesses slow and deliberate processes but also captures fast and effortless processing. However, this task has been widely used to measure regulatory processing in eating behavior research (e.g., Ames et al., 2014; Kakoschke et al., 2015; Veling et al., 2017; Jones et al., 2018). Nevertheless, future research could usefully consider other tasks to capture slow and deliberative processes. Relatedly, it should be noted that the attentional bias scores derived from the dot probe task included both negative (indicating an attentional avoidance) and positive (indicating attentional bias) scores. Although these were roughly equally distributed across our sample, future research in larger samples could seek to distinguish these two types of attentional processes and their relationships with food choice behavior.

Finally, the current study did not measure hunger. As hunger level could have influenced both the cognitive processing of food items (in the go/no-go and dot probe tasks) and the selection of energy-dense foods (in the food choice task), future research should endeavor to measure hunger at the start of the testing session.

### Theoretical and Clinical Implications

From a *theoretical* perspective, the current results may add to the dual-pathway model as a way of understanding intrapersonal determinants of eating behavior in adolescents. The current results should be considered preliminary and replicated in larger samples to substantiate *clinical implications*. In this context, the different results for the LOC-group and the NoLOC-group seem to indicate that the dual-pathway vulnerability does not apply to all adolescents. Specifically, for adolescents who experience LOC, the dual-pathway processes do not seem to have an added explanatory value in the context of food choices. However, for adolescents who do not experience LOC, screening efforts based on dual-pathway processes might be valuable. Adolescents with poor inhibitory control combined with low attentional bias might be particularly at risk of making unhealthy food choices.

## Conclusion

In conclusion, the current study did not find evidence for the dual-pathway perspective in predicting food choice behavior in adolescents. However, dual-pathway processes interacted with LOC in predicting food choice behavior. In adolescents with LOC, dual-pathway processes do not seem to have additional explanatory value. However, for adolescents who do not experience LOC, those with poor inhibitory control combined with low attentional bias might be particularly at risk of making unhealthy food choices. These findings provide an important next step in understanding food choice behavior in adolescents.

## Data Availability Statement

The raw data supporting the conclusions of this article will be made available by the authors, upon request.

## Ethics Statement

The studies involving human participants were reviewed and approved by the Ghent University Ethics Committee. Written informed consent to participate in this study was provided by the participants’ legal guardian/next of kin. Written informed consent was obtained from the minor(s)’ legal guardian/next of kin for the publication of any potentially identifiable images or data included in this article.

## Author Contributions

LG, EK, SV, LC, and EV designed the study and wrote the protocol. EV was responsible for data collection, under the supervision of LG. EV conducted the statistical analyses and wrote the first draft of the manuscript. All authors edited subsequent drafts and have approved the final manuscript.

## Conflict of Interest

The authors declare that the research was conducted in the absence of any commercial or financial relationships that could be construed as a potential conflict of interest.
